# Origin and Evolution of Enceladus’s Tidal Dissipation

**DOI:** 10.1007/s11214-023-01007-4

**Published:** 2023-10-06

**Authors:** Francis Nimmo, Marc Neveu, Carly Howett

**Affiliations:** 1 Dept. Earth and Planetary Sciences, University of California, Santa Cruz, CA 95064 USA; 2 Dept. Astronomy, University of Maryland, College Park, MD 20742 USA; 3 Planetary Environments Laboratory, NASA Goddard Space Flight Center, Greenbelt, MD 20771 USA; 4 Dept. Physics, Oxford University, Oxford, OX1 3PU UK

**Keywords:** Satellites of Saturn, Tidal heating, Astrometry, Resonance locking, Orbital evolution, Impact flux

## Abstract

Enceladus possesses a subsurface ocean beneath a conductive ice shell. Based on shell thickness models, the estimated total conductive heat loss from Enceladus is 25–40 GW; the measured heat output from the South Polar Terrain (SPT) is 4–19 GW. The present-day SPT heat flux is of order $100\text{ mW}\,\text{m}^{-2}$, comparable to estimated paleo-heat fluxes for other regions of Enceladus. These regions have nominal ages of about 2 Ga, but the estimates are uncertain because the impactor flux in the Saturnian system may not resemble that elsewhere. Enceladus’s measured rate of orbital expansion implies a low dissipation factor $Q_{p}$ for Saturn, with $Q_{p} \approx 3\times 10^{3}$ (neglecting the role of Dione). This value implies that Enceladus’s present-day equilibrium tidal heat production (roughly 50 GW, but with large uncertainties) is in approximate balance with its heat loss. If $Q_{p}$ is constant, Enceladus cannot be older than 1.5 Gyr (because otherwise it would have migrated more than is permissible). However, Saturn’s dissipation may be better described by the “resonance-locking” theory, in which case Enceladus’s orbit may have only evolved outwards by about 35% over the age of the Solar System. In the constant-$Q_{p}$ scenario, any ancient tidal heating events would have been too energetic to be consistent with the observations. Because resonance-locking makes capture into earlier mean-motion orbital resonances less likely, the inferred ancient heating episodes probably took place when the current orbital resonance was already established. In the resonance-locking scenario, tidal heating did not change significantly over time, allowing for a long-lived ocean and a relatively stable ice shell. If so, Enceladus is an attractive target for future exploration from a habitability standpoint.

## Introduction

One of the biggest surprises of the *Cassini* mission was the discovery of active venting and thermal anomalies at the South Pole of Enceladus (Porco et al. 2006; Spencer et al. 2006). Although it was immediately recognized that this activity was almost certainly due to tidal heating, the available tidal energy was initially thought to be insufficient (Meyer and Wisdom 2007). Later measurements of the orbital evolution of the Saturnian moons demonstrated that Saturn could in fact supply energy and angular momentum to these moons at a much higher rate than previously thought (Lainey et al. 2017, 2020; Jacobson 2022), thus potentially solving the Enceladus energy crisis. At about the same time, a combination of gravity (Iess et al. 2014), libration (Thomas et al. 2016) and chemical (Postberg et al. 2009) measurements revealed that Enceladus possessed a global ocean sandwiched between an ice shell and a low-density silicate core.

This paper will focus on the origin and evolution of tidal heating on Enceladus. There are two main reasons for doing so. First, apart from Io (Matsuyama et al. 2022), Enceladus is the best place in the solar system to understand the details of how tidal heating actually works, as well as its ultimate causes. Second, we need to understand how the balance between heat production and heat loss has evolved over time to understand the history of Enceladus’s ocean. A long-lived ocean is more likely to be a habitable environment than one that has only appeared recently.

Section 2 will summarize the pertinent observations, while Sect. 3 will discuss the theoretical aspects of the present-day tidal heating. Section 4 will make use of both observations and theory to discuss how tidal heating on Enceladus may have evolved with time, and Sect. 5 will summarize our conclusions.

This article overlaps with several other in this collection, notably those by Rhoden et al., Howett et al., Tobie et al., Ćuk et al. and Fuller et al. It also provides an update on Nimmo et al. (2018), which covered a similar set of topics.

## Observations

To understand the history of tidal heating on Enceladus, it is important to understand the general geologic setting and timing of events, as well as observational constraints on both its present-day and its ancient heat flux. Less obviously, it is also important to understand Enceladus’s orbital evolution, because this is driven by the tides in Saturn which are also the ultimate heat source for the moons. A brief discussion of Dione is also included, because in some cases all we can determine is the energy budget of the Dione-Enceladus pair (see Sect. 3.1).

### Global Geology and Ages

Broadly speaking, Enceladus can be divided into four regions (Crow-Willard and Pappalardo 2015; Patterson et al. 2018): the young, tectonized and geologically active south polar terrain (SPT); two tectonized regions of intermediate age, one centered on the trailing hemisphere (THT) and the other on the leading hemisphere (LHT); and the heavily cratered, ancient northern plains. The symmetry of this arrangement about the tidal axis is not understood, but the similarity of deformation at the LHT and the THT to that at the south pole suggests there were different episodes of heating at different locations on the satellite (Crow-Willard and Pappalardo 2015).

In principle, knowing the absolute ages of different terrains would provide a powerful constraint on Enceladus’s evolution. Unfortunately, the only current way of deriving such ages is by combining crater counts with a model impact flux. In the outer solar system, model impact fluxes for heliocentric impactors are poorly-calibrated (Zahnle et al. 2003). Furthermore, it is not entirely clear that the Saturn system has experienced the same impact history as the rest of the outer solar system (Ferguson et al. 2022), and indeed the system dynamics may have been perturbed only ∼100 Myr ago (Wisdom et al. 2022). Bearing these caveats in mind, crater counts (Kirchoff and Schenk 2009) show that the cratered plains on Enceladus have a median age around 4 Gyr, while the tectonized ridged plains are somewhat younger, with median ages of about 2 Gyr.^1^ The SPT region is very much younger, probably <1 Myr (Porco et al. 2006).

Although not as geologically active as Enceladus, Dione displays abundant tectonic features, both ridges and extensional troughs (Schenk et al. 2018). Based on cross-cutting relationships, some of the troughs are quite young, as is the large impact basin Evander (likely <2.5 Gyr and possibly <1 Gyr, (Kirchoff et al. 2018)). Although Dione’s heavily cratered plains are probably >4 Gyr old (Dones et al. 2009), its smooth plains are roughly 2 Gyr old (Kirchoff et al. 2018) and may have experienced cryovolcanism (Schenk et al. 2018). The overall impression is of a body that has experienced moderate geological activity over an extended period, or several discrete intervals.

### Direct Measurements of Present-Day Heat-Flux

Enceladus’s endogenic heat flux results in significantly increased surface temperatures near its South Pole, with the warmest temperatures occurring along the tiger stripes (Howett et al. 2011). The magnitude of these elevated temperatures has been determined using 4–5 $\mu $m data from Cassini’s Visible and Infrared Mapping Spectrometer (VIMS), and 7–1000 $\mu $m data from Cassini’s Composite Infrared Spectrometer (CIRS). Due to the non-linearity of the Planck function, CIRS is sensitive to a wider range of surface temperatures than VIMS, and thus has been the preferred dataset for such study.

To convert the measured surface temperatures into the desired estimate of heat flow, two questions arise: (1) what fraction of the observed emission is endogenic versus exogenic, and (2) how much of the surface is emitting at this level? A full description of how different studies have approached these questions, along with their relative advantages and disadvantages, may be found in the article by Howett et al. (this collection). However, a brief overview is provided here for context.

Initial 9–16 $\mu $m observations from CIRS were used to infer a heat flow of 5.8 ± 1.9 GW (Spencer et al. 2006), but these wavelengths are insensitive to any low-temperature (<65 K) endogenic emission. So a follow up study using 16–1000 $\mu $m observations that covered the entire SPT was conducted. These longer wavelengths are sensitive to lower temperatures (>30 K), but require the removal of the exogenic emission. This study inferred a much higher total heat flow of 15.9 ± 3.1 GW (Howett et al. 2011). Further work showed that the SPT’s four ≈150-km long tiger stripe fractures alone appear to be radiating 4.2 GW (Spencer et al. 2018), in which case the 35-km wide inter-tiger-stripe regions could be responsible for a few to roughly 10 GW of heat flow. Energy transport by latent heat from the tiger stripe plumes is small, roughly 0.5 GW (Ingersoll and Pankine 2010).

Thus, the total heat emitted by the south polar terrain is likely in the range 3.9–19 GW. The area south of $65^{\circ}\mathrm{S}$ constitutes 4.7% of the surface of Enceladus, so the corresponding average heat flux would be roughly $100\text{-}500\text{ mW}\,\text{m}^{-2}$. Even the low end of this range is comparable to terrestrial heat fluxes. Furthermore, it roughly matches the heat fluxes inferred from attempting to explain the “ropy” tectonic texture observed between the tiger stripes by compression (Bland et al. 2015). Even higher heat flux values (${\sim} 500\text{ mW}\,\text{m}^{-2}$) have been inferred for a region adjacent to the tiger stripes on the basis of microwave radiometry (Le Gall et al. 2017). However, this region does not show evidence of activity from either imaging or CIRS data, so the significance of this latter result is unclear. Geophysical constraints on ancient, as opposed to present-day, heat fluxes are discussed in Sect. 2.4 below.

### Estimates of Global Present-Day Heat Loss

As discussed above, the CIRS instrument is most sensitive to heat fluxes emitted by the hottest parts of Enceladus. However, we expect a low level of heat flow across the rest of the body, which will be undetectable by CIRS but nonetheless constitute a major fraction of the overall heat budget. Thus, the *measured* heat flow must be less than the total, global heat flow by an amount that is uncertain but could be large. To estimate this latter heat flow, we turn to models of global variations in shell thickness.

Enceladus’s ice shell is almost certainly conductive, because of the large shell thickness variations inferred primarily from gravity/topography measurements (see below). If the shell were convecting, these variations would have been smoothed out very rapidly by lateral flow of ice (Kamata and Nimmo 2017). Most recent works have also concluded that the amount of tidal heating in the ice shell is probably small compared to the global heat budget (e.g. Souček et al. 2019). If we assume a Cartesian, conductive shell and neglect internal heat production, then we can relate the local heat flux $F_{\mathrm{cond}}$ directly to the local shell thickness $d$:

1 $$ F_{\mathrm{cond}} = \frac{c}{d} \ln \left (\frac{T_{m}}{T_{s}}\right ) $$

Here the thermal conductivity is taken to vary as $c/T$, where $c=651\text{ W}/\text{m}$ is a constant (Petrenko and Whitworth 1999), $T_{s}$ is the surface temperature and $T_{m}$ the temperature at the base of the ice shell. Any tidal heating in the shell would be concentrated at its base and effectively reduce $T_{m}$. Given a map of global shell thickness variations, equation (1) can be integrated to determine the total conductive heat loss across the ice shell.

**Fig. 1:**
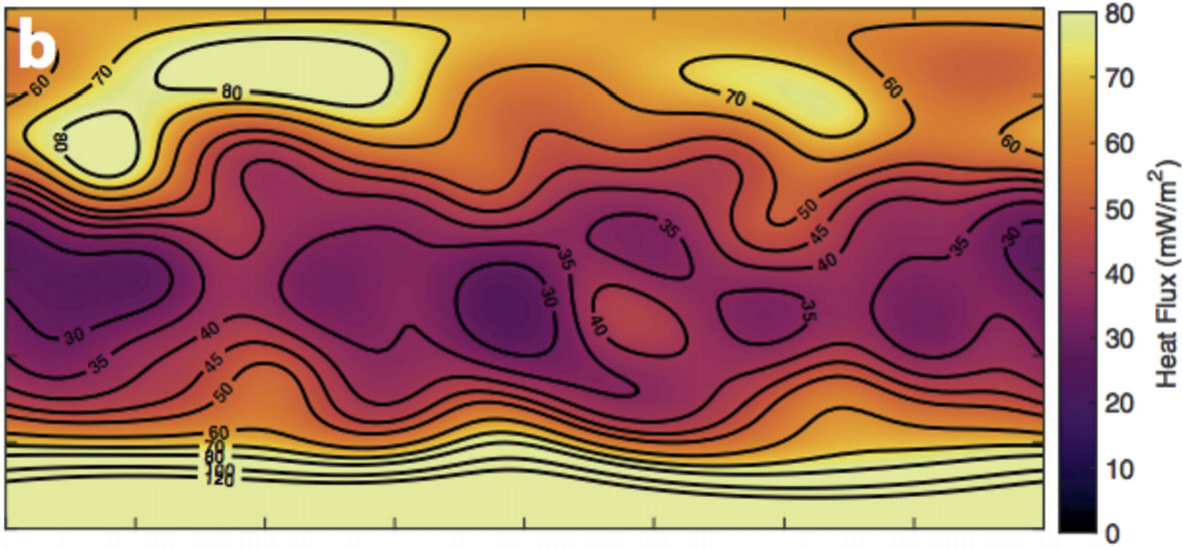
Spatial variation in conductive heat flux due to ice shell thickness variations, from Hemingway and Mittal (2019)

The ice shell thickness and its variations are determined by a combination of libration (Thomas et al. 2016) and gravity/topography (Iess et al. 2014) measurements, where for the latter Airy isostasy is assumed. A good summary of these approaches, which also calculates the global heat loss, is found in Hemingway and Mittal (2019). Figure 1 is taken from their paper and shows the inferred spatial variation in conductive heat loss. The high heat flow (${\approx} 160\text{ mW}\,\text{m}^{-2}$) at the South Pole is a consequence of the low shell thickness (≈5 km) there. Strikingly, the northern polar regions also exhibit high inferred heat fluxes, while in the equatorial regions heat fluxes are lower. This pattern is somewhat reminiscent of the heat flux variations expected for tidal heating in an ice shell (Ojakangas and Stevenson 1989).

Taking into account uncertainties in background ice shell thickness, density and so on, Hemingway and Mittal (2019) find a global conductive heat loss of 20–35 GW. This does not include the localized heat flow of ≈5 GW associated with the tiger stripes themselves, or the small contribution (0.5 GW) from advection of latent heat in the plumes. Thus, the global present-day heat loss at Enceladus is in the range 25–40 GW; Park et al. (submitted) find slightly smaller values (18–28 GW). In any event, these inferred heat flows provide one of our strongest observational constraints on the system.

At Dione, much less information is available. The available gravity and topography data are consistent with an ice shell of about 120 km thickness overlying an ocean, but non-ocean models are also permissible (Zannoni et al. 2020). In the ocean case, the total conductive heat flow would be roughly 25 GW, comparable to that estimated for Enceladus and an order of magnitude greater than the expected ≈2 GW of radiogenic heat production. Thus, the total heat loss in the Enceladus-Dione system might be as much as 65 GW.

### Estimates of Ancient Heat Fluxes

Even if an area is not being heated at the present day, it is sometimes possible to make estimates of ancient heat fluxes using geophysical techniques. These estimates, although uncertain, are useful because they allow us to piece together a history of tidal heating.

There are three principal techniques which have been used at Enceladus. The first is to infer the thickness of the elastic lithosphere at the time of deformation, and then convert that thickness to a heat flux. This technique was originally developed for the terrestrial oceanic lithosphere (Watts et al. 2013). The second is to model the viscoelastic relaxation of impact craters, and to infer the minimum heat flux required to have caused the observed relaxation, given some assumed heating duration. The third is to calculate the heat flux required to cause an instability of the correct (observed) wavelength to develop when a section of ice shell is subject to extension.

An example of this first approach is by Giese et al. (2008). These authors modeled the flexural topography of an ancient equatorial rift zone on the edge of the trailing hemisphere region and obtained an effective elastic thickness of 0.3 km. They used a yield-strength envelope approach (McNutt 1984) to convert this thickness to a heat flux in the range $200\text{-}270\text{ mW}\,\text{m}^{-2}$. The age of the deformation is uncertain, and model ages based on crater counts can differ by an order of magnitude depending on the assumptions made. Nonetheless, this heat flux probably records an event happening at least 1 Gyr ago (Giese et al. 2008).

Bland et al. (2012) looked at relaxed craters in two regions, one in the northern plains and one adjacent to the area investigated by Giese et al. (2008). Because of the small diameter of some of the relaxed craters, these authors found it was difficult to find conditions under which relaxation occurred. Even after appealing to a layer of insulating regolith to increase the effective surface temperature, they concluded that heat fluxes in excess of $150\text{ mW}\,\text{m}^{-2}$ would be required. Although the cratered terrains themselves are old, the heating event could have been significantly younger. The authors point out that heating can in some places apparently occur without accompanying deformation (unlike at the tiger stripes), and that areas of heating can be quite widespread.

Bland et al. (2007) examined two equatorial regions, including the one also analyzed by Giese et al. (2008). They argued that the ridges and troughs observed might be the result of an instability which can develop when the lithosphere undergoes extension. Although this constitutes a less direct estimate than the flexural approach, it produced comparable results: a heat flux of $110\text{-}220\text{ mW}\,\text{m}^{-2}$ and elastic thicknesses at the time of deformation of 0.4–1.4 km.

At Dione, a flexural study yielded an elastic thickness of 3.5 ± 1 km and a corresponding heat flux of $25\text{-}60\text{ mW}\,\text{m}^{-2}$ (Hammond et al. 2013), while impact crater relaxation studies yielded a lower bound on heat flux of $60\text{ mW}\,\text{m}^{-2}$ (White et al. 2017). These fluxes are an order of magnitude higher than the ${\approx} 6\text{ mW}\,\text{m}^{-2}$ expected if Dione currently possesses a deep ocean (see Sect. 2.3).

### Astrometry

The ultimate source of the energy being tidally dissipated in Enceladus is the rotational kinetic energy of Saturn. Some of this rotational energy and angular momentum gets transferred to the satellites via the tides that these bodies raise on Saturn. The rate at which the energy and angular momentum transfer happens depends on the so-called dissipation factor of Saturn, $Q_{p}$, which in general is frequency-dependent.

A combination of long-term telescopic observations and radio tracking of the *Cassini* spacecraft have allowed $Q_{p}$ to be determined at the frequencies of various satellites. For reasons explained below, it is more convenient to provide the results in terms of the outwards migration timescale of the satellite, $t_{\mathrm{tide}}$, rather than $Q_{p}$, where

2 $$ t_{\mathrm{tide}} = \left (\frac{1}{a} \frac{da}{dt}\right )^{-1} $$

where $a$ is the semi-major axis and $da/dt$ is the quantity that is measured by astrometry. An initial study which did not use spacecraft tracking yielded $t_{\mathrm{tide}}$ in the range 3–130 Gyr for Enceladus (Lainey et al. 2017), too uncertain to be very useful. A more recent study which incorporated tracking gave $t_{\mathrm{tide}} \approx 10\text{ Gyr}$, (Lainey et al. 2020). The equivalent value of $Q_{p}$ at Enceladus’s frequency for the latter study is $2030^{+3150}_{-1330}$, neglecting the influence of Dione (see equation (6)). An independent evaluation using both astrometry and tracking yielded a $Q_{p}$ at Enceladus’s frequency of 3136 ± 1317 (Jacobson 2022) and thus a $t_{\mathrm{tide}}$ of about 9 Gyr, compatible within error with the Lainey et al. (2020) result.^2^ The significance of these measurements will be discussed in much more detail below.

### Summary

Enceladus is emitting observable heat at a current rate of about 4–19 GW. The total conductive heat loss across the ice shell is about 25–40 GW, while Dione’s rate of heat loss, though uncertain, could be up to 25 GW. Present-day conductive heat losses at the SPT are around $160\text{ mW}\,\text{m}^{-2}$, which are compatible with estimates of ancient heat fluxes in both deformed equatorial and northern cratered regions. The age of these heating events is uncertain, but if current crater flux models are correct, the events are probably a few Gyr old. Astrometry has constrained the rate at which the inner satellites are moving outwards, and thus the rate at which Saturn is supplying energy and angular momentum to the satellites.

## Origin of Present-Day Dissipation

### Tidal and Equilibrium Heating

Tidal heating arises because, for a satellite in an eccentric orbit, the distance and direction to the primary is constantly changing and so too is the size of the tidal bulge (e.g. Murray and Dermott 1999). Unless the satellite is perfectly elastic, this continuous deformation will generate heat. The general expression for the rate of heat production if the eccentricity is small and the satellite’s spin and orbital period are synchronous is

3 $$ \dot{E} = \frac{21}{2} \frac{n^{5} R_{s}^{5} }{G} e^{2} \frac{k_{2s}}{Q_{s}} = 15\text{ GW}\ \frac{k_{2s}/Q_{s}}{0.01} $$

Here $n$ is the mean motion, $R_{s}$ the satellite radius, $G$ the gravitational constant, $e$ the eccentricity and $k_{2s}$ the so-called tidal Love number of the satellite, a measure of the response to the tidally-induced gravitational potential which indicates how deformable the satellite is. The quantity $Q_{s}$ is the quality factor of the satellite, a measure of how dissipative it is. Note that here we are neglecting any contribution from obliquity tides or forced librations.

Although we do not have a direct measurement of $k_{2s}/Q_{s}$ for Enceladus, we can estimate it as follows. If the tidal heat production is comparable to the heat loss (see Sect. 4.4), say 30 GW, then equation (3) yields a $k_{2s}/Q_{s}$ of 0.02. The actual value of this quantity will depend on the viscosity and rigidity structure of the body (see Tobie et al. this collection).

Equation (3) therefore shows that, in general, we need information about the internal structure of the satellite, as parameterized by $k_{2s}/Q_{s}$, to calculate the rate of tidal heating. However, for a satellite in a mean motion resonance with another satellite, this requirement can be avoided. This is extremely useful, as it makes our conclusions insensitive to poorly-known details about individual satellites’ structures.

A consequence of dissipative processes (Eq. (3)) is that the satellite’s eccentricity will damp over time, thus reducing the tidal heating rate (Murray and Dermott 1999). Thus, an isolated satellite is expected to evolve to a circular orbit over time, as is the case with Triton. However, for a satellite in a mean-motion resonance (MMR), the eccentricity is simultaneously being excited, as a result of the torques being applied on the satellites by the central planet (Murray and Dermott 1999). In the specific case of the Enceladus-Dione 2:1 resonance, it is Enceladus’s eccentricity that is getting excited (Meyer and Wisdom 2008b). There are thus competing eccentricity-exciting and eccentricity-damping mechanisms. As a result, the situation tends towards an equilibrium at which the eccentricity is constant. Furthermore, if this equilibrium is achieved, then the total tidal dissipation rate within the pair of satellites can be calculated without needing to know the satellite $k_{2}/Q$ values.

If the eccentricities of the satellites in resonance are small, this equilibrium tidal heating rate may be written (Meyer and Wisdom 2007)

4 $$ \dot{E}_{eq} = \frac{n_{0} T_{0}}{\sqrt{1-e_{0}^{2}}}+ \frac{n_{1} T_{1}}{\sqrt{1-e_{1}^{2}}} - \frac{T_{0} + T_{1}}{L_{0} + L_{1}} \left (\frac{GM_{p} m_{0}}{a_{0}} + \frac{GM_{p} m_{1}}{a_{1}}\right ) $$

where here subscripts refer to the inner (0) and outer (1) satellite, $n$ is the mean motion, $T$ is the torque on the satellite from Saturn, $L$ is the orbital angular momentum ($=m[GM_{p} a (1-e^{2})]^{1/2}$), $a$ is the semi-major axis and $m$ the satellite mass, assumed much smaller than Saturn’s mass $M_{p}$. An equivalent way of calculating the equilibrium heating rate was introduced by Fuller et al. (2016), but we use equation (4) in what follows because it makes the dependence on Dione torques more obvious. Below we take $e_{0} = 0.0047$ and $e_{1} = 0.0022$.

A disadvantage of this equation is that it does not specify in which body the tidal heating is happening. Because the current Enceladus-Dione resonance is exciting Enceladus’s eccentricity (Meyer and Wisdom 2008a), it has generally been assumed that the majority of heat production takes place in Enceladus. However, Dione also has a non-zero eccentricity and could thus (according to equation (3)) be dissipating energy too. It should also be noted that in some situations the instantaneous heating rate can oscillate about the equilibrium value (see Sect. 3.2 below).

The crucial quantity is the torque, which depends on the $Q_{p}$ of Saturn at the relevant frequency:

5 $$ T = \frac{3}{2} \frac{G m^{2} R_{p}^{5} k_{2p}}{a^{6} Q_{p}} $$

Here $R_{p}$ is Saturn’s radius (taken to be 58,232 km) and $k_{2p}$ its tidal Love number (taken here to be 0.34 (Gavrilov and Zharkov 1977) and independent of frequency). As will be seen below, whether there is a significant torque acting on Dione ($T_{1}$) has an important bearing on our conclusions. The importance of equation (4) is that it allows the total tidal heating in Enceladus and Dione to be estimated without knowing their internal structures, as long as $Q_{p}$ is known for both bodies.

The last piece of the puzzle is therefore to relate $Q_{p}$ to the astrometrically-derived orbital evolution timescale $t_{\mathrm{tide}}$. This is done using (Fuller et al. 2016):

6 $$ t_{\mathrm{tide}}=\frac{Q_{p}}{F}\frac{M_{p}}{m}\left ( \frac{a}{R_{p}}\right )^{5} \frac{1}{3 k_{2p} }n^{-1} \approx 3\text{ Myr} \frac{Q_{p}}{F} $$

There is thus a linear relationship between $Q_{p}$ and $t_{\mathrm{tide}}$. However, there is one complication, embedded in the factor $F$. For an isolated moon, $F=1$ and no further discussion is required. For a moon in a mean-motion resonance, it is *a priori* unclear whether the motion of the outer moon is as a direct result of torques being exerted on it by Saturn, or whether its motion is due to transfer of torques applied to the inner moon. If the latter case, then the outwards motion of the two moons will be reduced by some factor, because the torque on the inner moon is being applied to the combined angular momentum of both bodies. Conversely, in order to match a specified rate of outwards motion, the torque in the second case must be larger than in the first case, and thus the $Q_{p}$ must be smaller i.e. Saturn needs to be more dissipative. This additional factor is $F=\left [1 + L_{1}/L_{0}\right ]^{-1}$. Unfortunately, for the Enceladus-Dione case, $F\approx 0.07$ and so whether direct tidal torques on Dione ($T_{1}$) are important matters a great deal, both for deriving $Q_{p}$ and for determining the equilibrium heat flow (equation (4)). These issues are also discussed in Ćuk and El Moutamid (2023).

As explained in Sect. 4.1 below, Saturn torques most likely arise because there are peaks in Saturn’s dissipation spectrum occurring at the satellite frequency. For torques to be operating on both bodies simultaneously, there would have to be two resonant peaks in Saturn, at the synodic periods of Enceladus and Dione. Furthermore, the period ratio of these two peaks would have had to be maintained at 2:1 as Saturn evolved. Although theory suggests that neither eventuality is very likely, astrometry finds non-zero Saturn torques (i.e. a finite $Q_{p}$) on Dione (Lainey et al. 2020). As a result, the real situation is probably somewhere intermediate between the two cases presented in Table 1.

**Table 1:** Summary of Enceladus equilibrium heat flow calculations and Saturn dissipation factor at Enceladus frequency $Q_{p}$. Values for $t_{\mathrm{tide}}$ are based on (Lainey et al. 2020) and (Jacobson 2022); error bars in $Q_{p}$ are estimated based on the latter. $Q_{p}$ and $t_{\mathrm{tide}}$ are related via equation (6) and heat flow $\dot{E}$ is from equation (4). Note the large difference in whether direct torques on Dione ($T_{1}$) are included or not. The actual equilibrium heating rates are probably intermediate between these values (see text), and $Q_{p}$ at the frequencies of Enceladus and Dione may not be the same

$t_{\mathrm{tide}}$ (Gyr)	$Q_{p}$	*Ė* (GW)	Notes
9	190 ± 80	$190^{+150}_{-60}$	No direct torques on Dione (*F* = 0.07, $T_{1} = 0$)
9	2600 ± 1100	$7^{+5}_{-2}$	Direct torques on Dione (*F* = 1, $T_{1}\neq 0$)

Table 1 summarizes the ranges of possible equilibrium tidal heating rates for a value of $t_{\mathrm{tide}} = 9\text{ Gyr}$, consistent with both Lainey et al. (2020) and Jacobson (2022). A smaller $t_{\mathrm{tide}}$ would mean more rapid evolution, a lower $Q_{p}$ and higher heating rates. This Table illustrates the factor of ≈30 difference between neglecting and including the effects of torques on Dione. If the torques on Dione are zero, the equilibrium heating rate is in the range 130–350 GW. However, it is more likely that the torques on Dione contribute at some level. For instance, the lowest Dione torque case from Table 1 of Lainey et al. (2020) results in $Q_{p}$ values of roughly 700 and 4000 at Enceladus and Dione, respectively, and an equilibrium heating rate of roughly 50 GW. Most of this energy is presumably going to heat Enceladus rather than Dione.

### Periodic Heating

As explained above, for the Enceladus-Dione system there is a competition between eccentricity-forcing and eccentricity-damping which tends to produce a constant equilibrium eccentricity. However, this is not the only possible solution; as first pointed out by Ojakangas and Stevenson (1986), another possibility is that the eccentricity and heat production undergo periodic oscillations around their equilibrium values. This behaviour arises because of the feedbacks between the heating rate, the internal temperature (and thus viscosity) of the body, and the rate at which it dissipates energy. Periodic solutions are favored if the timescales of eccentricity damping and internal structure evolution are comparable.

Meyer and Wisdom (2008a) showed that the specific mechanism advocated by Ojakangas and Stevenson (1986) did not in fact produce periodic behaviour at Enceladus. However, Shoji et al. (2014) adopted a slightly different model set-up and found that for some parameter values periodic behavior could be generated. Oscillation periods were typically ≈100 Myr, but these authors assumed a constant $Q_{p}$ of 18,000, which results in low mean heating rates (1.1 or 2.4 GW, see (Meyer and Wisdom 2007)). A lower $Q_{p}$ would presumably have resulted in more rapid oscillations.

If it does experience oscillations, Enceladus could currently be in a state such that its instantaneous eccentricity and heating rate are below their equilibrium values, eccentricity damping is suppressed and the moon is moving deeper into the resonance. In principle, sufficiently good astrometric measurements should be able to determine whether the moon is moving into or out of the resonance; this would require a measurement of radial drift with a precision of better than 1 cm/yr. For Enceladus the competing effects of dissipation in Saturn and dissipation in the satellite are hard to disentangle (Lainey et al. 2020; Jacobson 2022).

### Location of Heating

Just as equation (4) does not specify how tidal heating is distributed between Enceladus or Dione, nor does it specify where within a particular body the heat is being produced. Most recent authors have concluded that heating in either the ice shell (e.g. Souček et al. 2019) or the ocean (e.g. Hay and Matsuyama 2019; Rovira-Navarro et al. 2019) are insufficient, although one recent work on internal waves in a stratified ocean argued that this heating mechanism could be important (Rovira-Navarro et al. 2023). Nonetheless, it appears likely that the bulk of heating occurs within the low-density core (Roberts 2015; Choblet et al. 2017). How exactly the core dissipates heat is an open question, although the specific mechanism of tidal fluid pumping (Rovira-Navarro et al. 2022; Kamata 2023) appears to be insufficient. The location of tidal heating is explored in more detail in the article by Tobie et al. (this collection).

### Comparison with Observations

Table 1 leaves us in a rather unsatisfactory state, as the high and low end of the tidal heat production estimates (5–350 GW) bracket the inferred heat loss rate of 25–65 GW (Sect. 2.3). The problem is that the direct Saturn torques on Dione are not known. The results of Lainey et al. (2020) suggest that a non-zero Dione torque does exist, but theoretical arguments suggest that it should not (see Sect. 3.1), and the uncertainties are large enough to also permit a zero-torque solution for Dione. Our current best guess is that the direct Saturn torques on Dione are small but non-zero, and that the heat production rate is intermediate, roughly 50 GW (Sect. 3.1). But the current uncertainties are large; reducing the uncertainties in these astrometric constraints would certainly improve our understanding of the problem.

At the low end of the heat production estimates shown in Table 1, Enceladus would be losing more heat than it is currently producing. The resulting ocean freezing timescale would be of order 10 Myr. Unless we are seeing Enceladus at a fortuitous time in its history, and the Saturn torques on Dione are large, this scenario seems unlikely.

At the high end, the heat production rate could be a factor of 6–16 times larger than the system conductive heat loss. An interesting consequence of this would be that Enceladus’s ocean would be melting quite rapidly: 100 GW of excess heating would thin the ice shell to zero in about 1 Myr! This scenario also seems unlikely, not least because a rapidly-thinning ice shell would produce a network of global compressional features (Nimmo 2004) which are simply not seen on the surface. One could argue that Enceladus is fortuitously at a high heat production point of an oscillating cycle, but the oscillation period would have to be short compared with 1 Myr. Or perhaps Dione is currently experiencing the bulk of the heating right now, but has not yet had time for the effects to reach the surface. Neither possibility seems likely.

If there is some torque contribution to Dione, the heat production rate (and inferred $Q_{p}$) will be intermediate between the high and low-end values of Table 1. As described in Sect. 3.1, the lowest Dione torque case examined by Lainey et al. (2020) produces a heat production rate of about 50 GW. This intermediate value would roughly balance the conductive heat loss rate, suggesting that Enceladus’s current structure (including its ocean) are potentially long-lived features. This result makes intuitive sense: if the heating rate exceeds the rate of heat loss, the ice shell will simply thin until thermal equilibrium is restored. Despite the uncertainties, it seems likely that for Enceladus, the present-day heat production and heat loss are roughly in balance. How that balance may have evolved over time is the subject of the next section.

## Evolution of Dissipation

### Semi-Major Axis Evolution

Recalling that $t_{\mathrm{tide}} = a / \frac{da}{dt}$, equation (6) can be integrated to determine how the semi-major axis $a$ varies with time, once $Q_{p}$ is specified.

In the simplest case of a constant $Q_{p}$, $a$ increases as roughly $t^{2/13}$ (Murray and Dermott 1999). More recently, it has been argued that $Q_{p}$ is unlikely to be constant, and that the orbital evolution timescale $t_{\mathrm{tide}}$ is instead ultimately controlled by some aspect of the evolution timescale of the planet. This idea is known as the “resonance-locking” hypothesis. It was first introduced by (Fuller et al. 2016) and has subsequently been further elucidated elsewhere (Nimmo et al. 2018; Lainey et al. 2020; Ćuk and El Moutamid 2023), including the article by Fuller et al. (this collection). An alternative formulation for calculating gas giant dissipation has been presented by Terquem (2021).

The first flavor of the resonance-locking hypothesis (Fuller et al. 2016) focuses on the evolving frequency of gravity modes in Saturn’s interior that are in resonance with tidal excitation by a satellite. This frequency changes on a timescale $t_{\alpha}$. In this case we have

7 $$ \frac{1}{a} \frac{da}{dt} = \frac{2}{3} \left [ \frac{\Omega _{p}}{n} \left ( \frac{1}{t_{\alpha}} - \frac{1}{t_{p}} \right ) - \frac{1}{t_{\alpha}}\right ] $$

Here $\Omega _{p}$ is Saturn’s spin frequency and $t_{p}$ is the spin evolution timescale. In this approach, if it is assumed that $t_{p} \rightarrow \infty $ then a simple analytical solution for $a(t)$ can be obtained (Nimmo et al. 2018). This solution typically produces satellites with diverging orbits, important for understanding their evolution through mean-motion resonances (see Sect. 4.3).

The second flavor (Lainey et al. 2020) is a special case of the first, in which the mode evolution timescale $t_{\alpha}$ is assumed proportional to the age of the planet $t$, such that

8 $$ \frac{1}{a} \frac{da}{dt} \approx \frac{B}{t} $$

where $B$ is a constant. If $t_{\mathrm{tide}} \approx 9\text{ Gyr}$ (Sect. 2.5), then $B\approx 1/2$, so we have $a$ evolving as roughly $t^{1/2}$. This version is more likely to be associated with inertial waves and results in satellites maintaining near-equal period ratios as they migrate outwards.

**Fig. 2:**
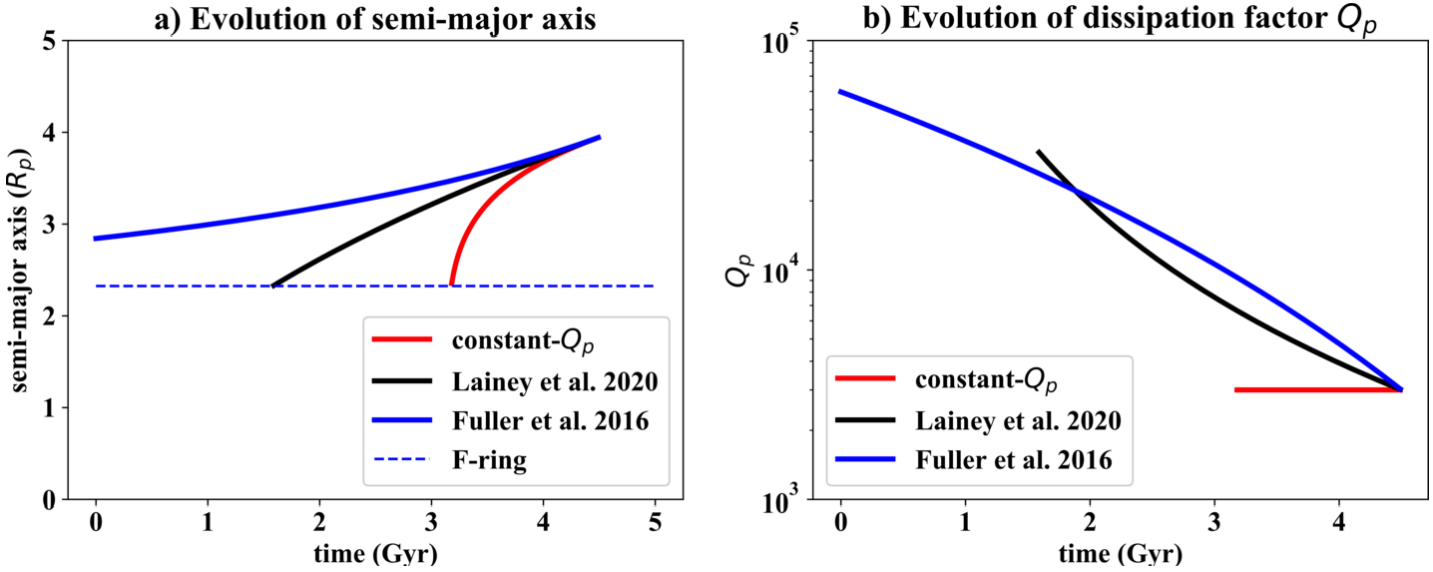
a) Evolution of Enceladus’s semi-major axis according to three different Saturn-$Q$ scenarios. The present-day evolution timescale $t_{\mathrm{tide}}$ is set to 9 Gyr in all cases. In the constant-$Q$ case, $Q_{p}$ is set to 3100. In the Lainey et al. (2020) case (equation (8)), $B = 0.49$. In the Fuller et al. (2016) case (equation (7)), $t_{\alpha} = 13\text{ Gyr}$. The location of the F-ring denotes where a satellite will be torn apart by tides. Here we neglect any influence of the mean motion resonance with Dione. b) Corresponding values of $Q_{p}$ as a function of time. Here we are taking $F = 1$ (equation (6)). Present day is at 4.5 Gyr

Figure 2a plots an example of the semi-major axis evolution of Enceladus under these three different scenarios, ignoring the effects of any mean-motion resonances. The present-day evolution timescale $t_{\mathrm{tide}}$ is set to 9 Gyr (cf. Table 1) in all three cases. For the constant-$Q_{p}$ model, Enceladus cannot be more than about 1.5 Gyr old. Conversely, using the Fuller et al. (2016) approach (equation (7)), Enceladus has only increased its semi-major axis by about 35% over 4.5 Gyr. Lastly, the Lainey et al. (2020) approach (equation (8)) yields an intermediate result with an Enceladus that could be as old as 3 Gyr. An important consequence of the resonance-locking hypotheses is that a young Enceladus is *not* required, despite its relatively rapid present-day outwards migration.

Figure 2b plots the corresponding evolution of $Q_{p}$. In both the Lainey et al. (2020) and Fuller et al. (2016) scenarios, $Q_{p}$ increases with decreasing semi-major axis. This is important when considering past rates of tidal heating (see Sect. 4.2).

It should be emphasized that the results shown in Fig. 2 are subject to large uncertainties. Not only is the present-day outwards evolution rate of Enceladus quite uncertain, but the resonance-locking models make assumptions about the rate at which the internal structure of Saturn is changing which are likely to become progressively more inaccurate at earlier times. Furthermore, for Fig. 2b the $Q_{p}$ values derived depend on whether torques from Dione are included or not (equation (6)), which adds an additional factor of 30 uncertainty. Finally, these calculations are only relevant while the Enceladus-Dione mean motion resonance is operating, and of course the age of that resonance is not known (see Sect. 4.3).

### Tidal Heating Evolution

**Fig. 3:**
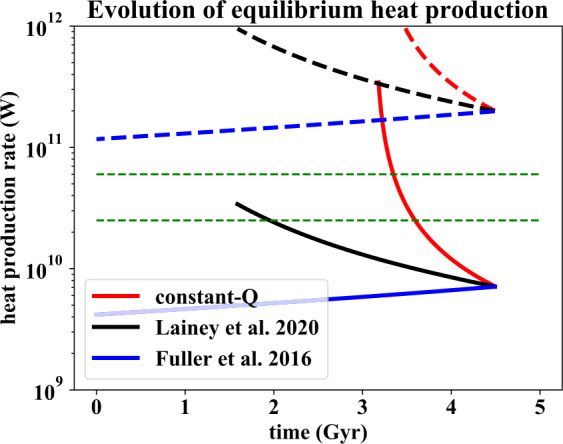
Evolution of equilibrium heat production rate as a function of time, using the same parameters as for Fig. 2. Solid lines are calculated using equation (4) with $F = 1$, while dashed lines use $F = 0.07$, to reflect the effect of uncertainties in the role of Dione torques. The true values will lie between the two extremes. The horizontal dashed green lines denote the estimated heat loss rate of the Enceladus-Dione system (25–65 GW)

Figure 3 uses the same values as Fig. 2 to plot how equilibrium tidal heat production has evolved with time. For each of the three scenarios, lower (solid line) and upper (dashed line) values are plotted depending on whether torques on Dione are included in the calculations of $Q_{p}$ (equation (6)) and $\dot{E}$ (equation (4)). As discussed above (Sect. 3.1) the true value will be somewhere between the two extremes. The green dashed lines represent the likely range of heat loss (25–65 GW) in the Enceladus-Dione system.

Even though the heat flow calculations only apply during the Enceladus-Dione MMR, it is clear that either constant-$Q$ model would rapidly run into problems at earlier times, generating Io-like ($1\text{ W}\,\text{m}^{-2}$) heat fluxes. Unless the resonance was encountered recently, a constant-$Q_{p}$ scenario can be ruled out. Conversely, the Lainey et al. (2020) heat flow increases more modestly with decreasing age, while the Fuller et al. (2016) model actually shows a small decrease at earlier times. The implications of these results will be discussed further in Sect. 4.4.

Although the values shown in Fig. 3 are specific to the Enceladus-Dione 2:1 resonance, equilibrium tidal heating rates for other resonances are not very different (Meyer and Wisdom 2007) and would show qualitatively the same behavior. Thus, if Enceladus encountered ancient resonances prior to its current one, we would expect heating rates similar to those in Table 1 and Fig. 3.

### Constraints on Orbital Evolution

The considerable uncertainties in Enceladus’s evolutionary path, which arise from the unknown nature and evolution of tidal dissipation inside Saturn and the uncertain present-day rates of orbital expansion of Enceladus and Dione, bring about a need for constraints on the past orbital evolution of these moons. These constraints fall into two main categories: orbital characteristics; and inferences of past heating episodes. As noted in Sect. 2.1, age constraints are based on impactor flux models that may not be relevant to the Saturn system, so their usefulness is limited. Further discussion on the orbital constraints may be found in the articles by Ćuk et al. (this collection) and the paper by Ćuk and El Moutamid (2023).

#### Orbital Characteristics

There are three major obstacles in obtaining constraints based on orbital characteristics. First, due to the overall compactness of Saturn’s mid-sized moon system, the orbital evolution of Enceladus is not solely tied to that of Dione, but also to those of the other moons in that system: Mimas, Tethys, and Rhea (Neveu and Rhoden 2019). All five moons are packed between 3 and 9 Saturn radii, which implies a high potential for encountering orbital resonances throughout their history. Titan’s larger distance from Saturn makes it less likely that it has entered orbital resonances with the inner moons that played a major role in their orbital evolution.

Second, unlike secular (nonresonant) orbital evolution, the outcome of orbital (e.g., mean motion) resonances can be stochastic (e.g. Ćuk et al. 2020). This makes it difficult to constrain the orbital properties (semi-major axes, eccentricities, inclinations) of two satellites before a resonance is encountered even if these are known after the resonance is exited, and vice versa.

The third obstacle is a consequence of the first two: the age of the moons is unknown. In such a compact system where orbital semi-major axes and eccentricities are prone to large relative changes due to gravitational interactions between moons (Malhotra 1998), collision, ejection, and tidal disruption events (if a moon enters Saturn’s Roche limit) are more likely (e.g. Nakajima et al. 2019). Saturn’s ring-moons and moonlets, whose sizes increase with increasing distance from Saturn (Tiscareno et al. 2006) testify to ongoing accretion in the system (Charnoz et al. 2010). The rings, whose mass is 40% that of Mimas, have been argued to be much younger than the age of the solar system (Iess et al. 2019; Wisdom et al. 2022). If valid (see Crida et al. 2019, for counter-arguments), this inference suggests the geologically recent collision or disruption of one or more moons (e.g. Wisdom et al. 2022). More generally, material may have been cycled between forming mid-sized moons like Enceladus and Dione and forming rings several times since the formation of Saturn at the dawn of the solar system.

These obstacles notwithstanding, the secular orbital evolution of moons due to their tidal interaction with Saturn (Sect. 4.1) indicates that the orbits of moons expand with time. Superimposed on this interaction are moon-ring interactions, which result in comparatively fast outward migration of moons whose orbital semi-major axis is less than $R_{\text{F-ring}} \times 2^{2/3} = 222{,}000\text{ km}$ (Charnoz et al. 2011), where $R_{\text{F-ring}}$ is the radius of the F ring (Fig. 2a). For example, the orbit of Mimas, which is currently within this zone, would have expanded from an orbit twice as close to the rings in just 0.7 Gyr from moon-ring interactions alone if the rings predate Mimas (Neveu and Rhoden 2019). This is comparable to a constant-$Q_{p}$ rate of orbital expansion and much faster than that due to resonance locking at these orbital distances (Fig. 2a). Finally, a moon’s orbit may contract if tidal dissipation inside the moon is high; this effect complicates interpretation of Enceladus’s astrometry (see Sect. 3.1) as well as making its orbital evolution more uncertain.

The reliability of the above constraints then rests on the propensity of moons to enter orbital resonances. In the case of mean-motion resonances (MMRs), this propensity depends significantly on the mechanism of orbital expansion; only if satellites are on converging orbits is capture into an MMR possible (Murray and Dermott 1999). In the constant-$Q_{p}$ picture, numerous converging resonances are encountered, making the moons’ shorter orbital history unpredictable. At the other extreme, the special case of resonance locking suggested by the observations of Lainey et al. (2020) (equation (8)) preserves semi-major axis ratios between moons such that mean-motion resonances are never encountered unless two moons formed in resonance. Generally, the propensity for capture into mean-motion resonances is much lower under the resonance-locking hypothesis than if $Q_{p}$ is constant. But, as explained above (Sect. 4.2), we regard the constant-$Q_{p}$ case as unlikely.

The present-day existence of the Enceladus-Dione and Mimas-Tethys mean-motion resonances suggests that some evolution of orbits on converging tracks is likely to have occurred. For instance, Mimas’s current eccentricity and Tethys’s current inclination are both high, but are not being substantially excited at present; this suggests that these bodies experienced some other kind of resonance in the past (see Ćuk et al.). A past 3:2 Mimas:Enceladus resonance might have excited the eccentricity of either Mimas (Meyer and Wisdom 2008a; Tian and Nimmo 2020) or Enceladus (El Moutamid et al. 2019); the latter possibility might explain evidence for older heating episodes (Sect. 2.4). Other resonances that may have affected the inner Saturnian moons are less relevant to Enceladus, and are discussed in more detail in the article by Ćuk et al.

#### Past Heating Events

The ongoing Enceladus-Dione and Mimas-Tethys mean-motion resonances, as well as evidence for past high heat flow on Enceladus (Sect. 2.4), Tethys (White et al. 2017) and Dione (Hammond et al. 2013), suggest that the non-converging orbits implied by equation (8) may not fully describe the moons’ orbital evolution, despite being compatible with observed rates of orbital expansion within uncertainties. In the specific case of Enceladus, the inferred ancient heat fluxes (Sect. 2.4) are comparable to those measured at the present day, and are consistent with heating during previous resonances. For instance, with $Q_{p} = 2000$ the Mimas:Enceladus 3:2 resonance would have produced an equilibrium heating rate of 8.6 GW (Tian and Nimmo 2020).

In addition to improving the astrometry of the mid-sized moons, a path forward in obtaining better constraints may lie in meshing the relative chronologies of (1) resonances encountered for a given expansion mechanism (see Ćuk et al. this collection) and (2) episodes of past high heat flow from geological evidence at all five mid-sized moons, together with detailed $N$-body orbital evolution simulations of relevant resonances.

### Comparison with Observations

Very little is known for sure about the orbital evolution of Enceladus and the other Saturnian moons. However, the constant-$Q_{p}$ and resonance-locking hypotheses make predictions which are very different and can potentially be tested.

The most important difference between the two models is the predicted ancient heat fluxes (Fig. 3). Assuming an MMR is operating, the predicted heat fluxes in the constant-$Q_{p}$ model rapidly reach values in excess of $1\text{ W}\,\text{m}^{-2}$ going back in time, implying a mean ice shell thickness of <1 km. Such a shell thickness is inconsistent with the existence of large, relatively unrelaxed craters, and is hard to reconcile with the apparently ancient nature of some parts of Enceladus. Unless the MMR was established recently - close to the present-day semi-major axis - the constant-$Q_{p}$ mechanism does not agree with the observational constraints.

In contrast, in the resonance-locking case the predicted heating rates vary only weakly with time and the intermediate value of 50 GW derived earlier (Sect. 3.1) is roughly consistent with the present-day estimated heat loss and estimates of past heating. In this scenario, the ice shell could have experienced either modest thickening or thinning with time. Shell thinning would lead to compression, while shell thickening would lead to extension (e.g. Nimmo 2004). On Dione there is plentiful evidence for extension and essentially none for compression (Schenk et al. 2018), but on Enceladus the tectonic picture is much more complicated (Patterson et al. 2018). Both compressional and extensional features have been inferred, but the amount of global extension or contraction is unknown. This absence of a clear signal may simply be telling us that the ice shell thickness has stayed roughly constant with time, and that other sources of stress, such as diurnal tidal stresses, non-synchronous rotation or true polar wander, are dominating the tectonic record. A roughly constant ice shell thickness would be consistent with the resonance-locking theory.

The constant-$Q_{p}$ model for Enceladus implies that Enceladus could be at most 1.5 Gyr old (Fig. 2). On the face of it, this is inconsistent with the crater-derived terrain ages (Sect. 2.1). However, these ages were derived using an assumed heliocentric impactor flux model which may not be appropriate to the Saturn system. The identification of a population of apparently planetocentric impactors (Ferguson et al. 2022) suggests that the heliocentric model is probably overestimating the age of Saturnian satellite terrains. Thus, it is not yet clear whether the “young Enceladus” paradigm can be ruled out on the basis of crater statistics.

The fact that some regions of Enceladus appear to have experienced ancient heating events could be interpreted in one of two ways. Either the spatial location of the current heating has shifted over time, perhaps due to true polar wander (Nimmo and Pappalardo 2006) or some kind of runaway feedback (Kang and Flierl 2020). Or the earlier events were caused by a different MMR, prior to capture into the current resonance. It is hard to distinguish between these two possibilities, because both the cratering timescale (see Sect. 2.1) and the orbital evolution timescale are very uncertain. The constant-$Q_{p}$ model produces larger semi-major axis evolution and would thus imply more resonance crossings. However, the large orbital expansion would mean that earlier heating events would be much more energetic (Fig. 3), which is hard to reconcile with the fact that ancient estimated heat fluxes are similar to the present-day values (Sect. 2.4). In contrast, the resonance-locking model is less likely to result in resonant capture (Sect. 4.3) and would favour persistence of the current resonance and heating.

In principle, the composition of Enceladus’s ocean might be used to place a constraint on how long it has been in existence. The problem is that the reaction rates of many silicates and organic compounds tend to be very fast compared to likely ocean lifetimes (e.g. Truong et al. 2019; Hao et al. 2022), so the ocean composition is controlled primarily not by reaction times but by the water-rock ratio, which depends on unknown details of the silicate core of Enceladus (e.g. how permeability varies with depth). The $\mathrm{H}_{2}$ and $\mathrm{CH}_{4}$ detected in Enceladus’s plumes are plausibly the result of hydrothermal reactions with the core (Daval et al. 2022; Etiope and Sherwood Lollar 2013), but at least for $\mathrm{H}_{2}$ such reactions are expected to be complete within a few hundred Myr. How to reconcile this inference with Enceladus’s apparently protracted history of heating is not clear.

## Summary & Future Work

Enceladus maintains a subsurface ocean beneath an icy shell, and Dione may have an ocean too. The estimated heat loss from the present-day Enceladus-Dione system based on conductive ice shell models is 25–65 GW (Sect. 2.3). This is a few to ten times larger than the measured heat output at the SPT of 4–19 GW (Sect. 2.2), because most of the heat is escaping at a low rate that makes it difficult to distinguish from the background insolation. Different regions of Enceladus appear to have experienced elevated heat fluxes (of order $100\text{ mW}\,\text{m}^{-2}$) in the past, comparable to those inferred for the present-day SPT (Sect. 2.4). These heating events are probably a few Gyr old, but the timing (which is based on crater counts) is uncertain. Dione also appears to have experienced heating episodes in the past, though perhaps of a lower amplitude.

Astrometry shows that Enceladus’s orbit has expanded outwards at an unexpectedly rapid rate (Sect. 2.5). This observation implies high levels of dissipation within Saturn, and a low dissipation factor $Q_{p}$. If $Q_{p}$ has stayed constant with time, Enceladus must be young, less than about 1.5 Gyr old. On the other hand, if the “resonance-locking” theory is correct (Sect. 4.1), $Q_{p}$ was larger in the past and Enceladus’s orbit may have expanded by only 35% over 4.5 Gyr. Because they are based on specific impactor flux models, crater-derived age estimates cannot distinguish between these two possibilities.

At the present day, Enceladus is in an MMR with Dione and there appears to be a rough balance between the amount of tidal heat being generated, and the amount of heat being lost (Sect. 4.2). If the constant-$Q_{p}$ model is correct, this resonance can only have been established recently, as equilibrium heating rates increase very markedly with decreasing semi-major axis (Fig. 3). This fact also makes the occupation of earlier MMRs in the constant-$Q_{p}$ case difficult to reconcile with the geophysical evidence (Sect. 2.4). In contrast, the resonance-lock model predicts roughly constant heat fluxes and a long-lived ocean, and is less likely to have caused capture into prior MMRs (Sect. 4.3).

Our understanding of Enceladus’s thermal and orbital evolution is still very much in its infancy. The constant-$Q_{p}$ model is hard to reconcile with our current knowledge of Enceladus’s heating history (Sect. 4.4), but we are hampered by very uncertain dating techniques, and the considerable dynamical complexity of the Saturnian satellites. A putative, recent catastrophic event (Wisdom et al. 2022) further complicates matters.

One important step forwards would be to better understand the cratering history of the inner Saturnian satellites. The identification of a population of planetocentric impactors (Ferguson et al. 2022) is important and should be followed up, along with improved impactor flux models. The likely implications of the recent disruption of a satellite for the impactor population should likewise be explored. Thermal-orbital evolution models (e.g. Neveu and Rhoden 2019) constrained by geophysical inferences could be pursued, but our current ignorance of how to describe the $Q_{p}$ of Saturn means that firm conclusions will be elusive.

Further advances may require observations of Enceladus by new spacecraft, as advocated by the 2023–2032 Planetary Science and Astrobiology Decadal Survey. In particular, such a spacecraft (e.g. Ermakov et al. 2021) would greatly improve our astrometric knowledge, determining $Q_{p}$ with much better precision. It would also provide a direct measurement of the $k_{2}/Q$ of Enceladus. This would in turn allow a calculation of the heat production in Enceladus (Eq. (3)), providing a direct test of the concept of equilibrium heating (Sect. 3.1). Improved global heat flow measurements (from infra-red observations) would allow a direct comparison between heat production and heat loss. Measurement of noble gas abundances in the plumes would help determine the history of outgassing and thermal activity (Truong et al. 2022).

Although there are considerable uncertainties, the resonance-locking model currently appears more consistent with observations than the constant-$Q_{p}$ model. This implies that Enceladus is old, with a long-lived ocean, making it an attractive astrobiological target. While the next mission to Enceladus may be focused on habitability, we expect that it will also solve many of the puzzles outlined in this article, and we look forward to seeing the results.

## Footnotes

1 Here we are using model A of (Zahnle et al. 2003), because this model is heavily favored based on crater counts in the Pluto/Charon system (Singer et al. 2019).

2 One important difference in the results is that Lainey et al. (2020) find that Enceladus is currently moving outwards, while Jacobson (pers. comm.) finds that it is moving inwards. Because dissipation in Saturn and Enceladus respectively increase and decrease the semi-major axis, the *net* motion of Enceladus is hard to determine and increases uncertainty in $Q_{p}$.
